# Monitoring Detrusor Oxygenation and Hemodynamics Noninvasively during Dysfunctional Voiding

**DOI:** 10.1155/2012/676303

**Published:** 2012-09-18

**Authors:** Andrew J. Macnab, Lynn S. Stothers, Babak Shadgan

**Affiliations:** ^1^Near Infrared Spectroscopy Research Group, Department of Urology, Faculty of Medicine, University of British Columbia and UBC Hospital Bladder Care Centre, Unit IB—Room F329, 221 Wesbrook Mall, Vancouver, BC, Canada V6T 1Z3; ^2^Stellenbosch Institute for Advanced Study, Wallenberg Research Centre, 10 Marais Street, Stellenbosch 7600, South Africa

## Abstract

The current literature indicates that lower urinary tract symptoms (LUTSs) related to benign prostatic hyperplasia (BPH) have a heterogeneous pathophysiology. Pressure flow studies (UDSs) remain the gold standard evaluation methodology for such patients. However, as the function of the detrusor muscle depends on its vasculature and perfusion, the underlying causes of LUTS likely include abnormalities of detrusor oxygenation and hemodynamics, and available treatment options include agents thought to act on the detrusor smooth muscle and/or vasculature. Hence, near infrared spectroscopy (NIRS), an established optical methodology for monitoring changes in tissue oxygenation and hemodynamics, has relevance as a means of expanding knowledge related to the pathophysiology of BPH and potential treatment options. This methodological report describes how to conduct simultaneous NIRS monitoring of detrusor oxygenation and hemodynamics during UDS, outlines the clinical implications and practical applications of NIRS, explains the principles of physiologic interpretation of NIRS voiding data, and proposes an exploratory hypothesis that the pathophysiological causes underlying LUTS include detrusor dysfunction due to an abnormal hemodynamic response or the onset of oxygen debt during voiding.

## 1. Introduction

Near infrared spectroscopy (NIRS) is an established noninvasive technique for monitoring changes in tissue oxygenation and hemodynamics in real time [1–4]. Simultaneous monitoring with pressure flow studies is recognized to add physiologic data of relevance in the evaluation of voiding dysfunction [5, 6]. A series of NIRS monitoring studies now suggest that during voiding changes can be detected in the detrusor microcirculation, which imply that abnormalities in hemodynamics or oxygen supply and demand occur in association with symptoms generated in several different situations, where there is voiding dysfunction [6–14]. These include bladder outlet obstruction (BOO) in males, nonneurogenic lower urinary tract dysfunction (NLUTD) in children, detrusor over activity (DO) in patients with neurogenic bladders due to spinal cord injury, and DO and over active bladder (OAB) in adult women. However, “More studies are needed to further define and validate uses for NIRS in urology” [5, 6]. Hence, this methodology report describes how urologists can conduct simultaneous NIRS monitoring of detrusor oxygenation and hemodynamics during UDS and summarizes the principles underlying physiologic interpretation of NIRS voiding data. Based on the published studies cited and literature referenced, the clinical implications of applying NIRS to study voiding dysfunction are outlined, the limitations of the technique discussed, and an exploratory hypothesis proposed that LUTS can result when an abnormal hemodynamic response or the onset of oxygen debt occurs in the detrusor during voiding. 

It is necessary to recognize when reviewing NIRS urologic research done to date [6–14], and in conducting further studies where NIRS monitoring is combined with UDS, that several different causal aetiologies are recognized to occur in conditions associated with lower urinary tract symptoms [15, 16] and that the parameters measured by each technique are very different; pressure and flow in UDS, and changes in the concentration of oxygenated and deoxygenated hemoglobin with NIRS (from which variations in oxygen supply and demand and the hemodynamics of the detrusor microcirculation are inferred). 

The clinical relevance of NIRS is the potential benefit provided by this additional physiologic information generated where voiding dysfunction is due to abnormalities that negatively impact the detrusor microcirculation. In a practical context, this information can contribute to a greater understanding of the pathologies causing LUTS, add diagnostic potential currently lacking, and contribute to selection and efficacy evaluation of specific therapeutic agents. However, correlation between NIRS parameters and UDS measurements can only be expected when pathology affecting detrusor hemodynamics or oxygen supply and demand underlies the patient's symptoms and pressure and flow data. This applies, for example, in the context NIRS studies in subjects with benign prostatic hyperplasia (BPH). Three studies have found comparable sensitivity and specificity when comparing NIRS with UDS diagnosis for BOO [7, 14, 17]; one did not [6]. However, patients with BPH are a particularly relevant population for application of NIRS monitoring as different causal pathologies can affect the structure, contractile properties, and vascular supply of the detrusor, and hence, as the pathologic mechanism responsible for voiding dysfunction and LUTS developing varies, there are several plausible sites of action for therapeutic interventions currently recommended [16, 18]. 

However, “at this stage NIRS does not seem ready to replace standard urodynamic testing,” [5, 19] and more studies are required to establish the value of NIRS in clinical practice [6, 19] and where the technique can contribute effectively and reliably in the evaluation of voiding dysfunction.

Hence this paper summarizes the methodology and principles underlying NIRS bladder monitoring to enable urologists to contribute the study data and discussion required for the clinical relevance of the technique to be established. 

## 2. Methods of Measurement

### 2.1. Physics Principles

NIRS shares similarities to oximetry, the most familiar form of optical monitoring of tissue oxygenation. Both are noninvasive and use energy in the form of light shone into the tissues through the skin to detect changes in the concentration of hemoglobin in real time. As in published descriptions of the fundamentals and applications of the technology to study muscle [1, 2, 4], brain [1, 3], and the bladder [11, 20], NIRS uses multiple wavelengths of near infrared (NIR) light, which penetrate into tissue, scatter, and depending on NIR wavelength are variably absorbed by oxygenated (O_2_Hb) and deoxygenated hemoglobin (HHb) in the field. From differences between the light emitted and detected returning to the sensor on the skin the concentrations of O_2_Hb, HHb, and the sum of the two, total hemoglobin (tHb), can be monitored. Displayed graphically the trends and relative changes from baseline in these parameters are used to infer variations in tissue blood volume, the provision of oxygenated blood, and the balance of oxygen supply and demand.

### 2.2. Study Technique

NIRS bladder studies are done via a patient interface that contains NIR light emitter and detector components. In laser powered NIRS instruments combined with UDS equipment, this interface is a self-adhesive patch which is placed on the abdominal skin over the anterior wall of the bladder 2 cm above the symphysis pubis and across the midline [21]. This positions the light emitter and photodiode detector over the anterior wall of the bladder. The depth of penetration of NIR photons is a factor of the distance between the instrument's emitter and detector; a 4 cm separation is most common for measurements of the anterior bladder wall and enables the detrusor to be interrogated in all but the most obese subjects [11, 13, 20, 22].

NIRS monitoring can be done during free uroflowmetry or simultaneously during invasive cystometry and pressure flow studies (UDS) conducted according to International Continence Society guidelines [6]. A unique feature of NIRS is the ability to collect data during natural filling and during voiding. This provides information on hemodynamics, which is not otherwise available without the presence of a catheter. Likewise, during voiding NIRS can provide physiologic data without the presence of a catheter. In all situations, both with and without a catheter the NIRS data collected are real-time changes in the concentration of O_2_Hb and HHb, and tHb. It is important that NIRS baseline data are collected (usually for 30 seconds) before patients are given permission to void in the setting of noninvasive flow studies, and bladder sensations related to urge, urgency, and capacity are documented on the tracing [6, 21]. NIRS data changes may be evident following the command for permission to void and before uroflow is recorded in the flow meter.

### 2.3. Data Validity

Confirmation that a NIRS device located on the abdominal skin over the bladder is detecting changes can be made by testing the effect of cough and Valsalva maneuver [12]; simultaneous ultrasound confirms the proximity of the device and the anterior bladder wall during voiding [22], and physiologic data related to the detrusor are only obtained from an NIRS interface located over the bladder and during events in the voiding cycle [20, 21]. Patient movement and abdominal straining are ideally restricted during study [12]. The potential contribution of movement artefact can be evaluated from the UDS record, from episodes of increased abdominal pressure and surface electromyogram (EMG) monitoring of abdominal wall muscle activity. The rationale for NIRS derived changes in haemoglobin concentration during voiding reflecting changes occurring in the detrusor microcirculation has been reviewed previously [7, 9, 11, 20]. 

### 2.4. Patient Selection

Published clinical studies [6–14] describe the cohorts of patients selected who range in age from 5 to 78 years [9]. In practice almost all patients with voiding dysfunction can be studied because of the optical nature of the technology, and NIRS monitoring is readily accepted by patients because of its noninvasive nature [11]. High Body mass Index (BMI) is a potential limiting factor as NIR light penetration is influenced by body fat [1, 23]. A BMI in excess of 30 kg/m^2^ has been identified to preclude measurement [13], and haematuria is a contraindication due to the absorption of light by hemoglobin in the urine.

## 3. Data Analysis Principles 

The changes in NIRS parameters most relevant physiologically are as follows.

1. The trend in total hemoglobin concentration (tHb), from which hemodynamic variations can be inferred. A positive or negative trend reflecting an increase or decrease in blood volume, respectively.
2. Changes in the concentration of O_2_Hb and HHb, which add information to any variation in blood volume, and in the presence of hemodynamic stability, allow variations in oxygen supply and demand to be inferred. Stable or increasing O_2_Hb associated with comparable changes in HHb implies a balance in oxygen supply and demand, whereas an increase in HHb with no rise in O_2_Hb reflects an imbalance and when associated with a simultaneous fall in O_2_Hb indicates the onset of oxygen debt.

**Figure 1:**
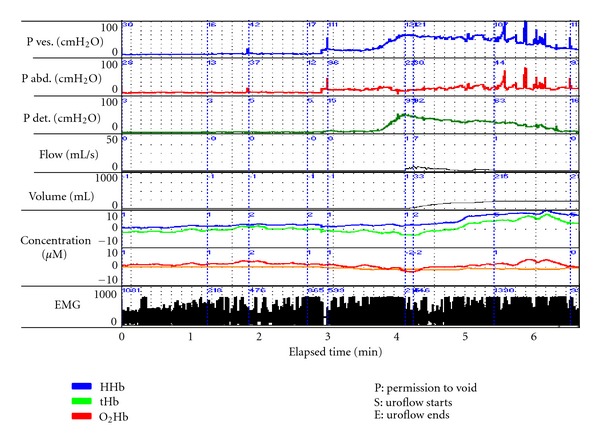
A composite graph of UDS data with NIRS parameters total hemoglobin (tHb) deoxygenated hemoglobin (HHb) and oxygenated hemoglobin (O_2_Hb) and EMG in a 64-year-old man. He had a 5-year history of increasing obstructive LUTS, had failed medical therapy, and complained of difficulty initiating urination, a weak stream, and slow flow. As Pdet increases prior to uroflow, there is a decrease in O_2_Hb reflected by a fall in tHb and HHb begins to rise. During uroflow, this positive trend in HHb increases, some rise in O_2_Hb is evident following Qmax but the associated rise in tHb predominantly reflects a greater rise in HHb concentration. This implies an imbalance in oxygen supply and demand during voiding.

**Figure 2:**
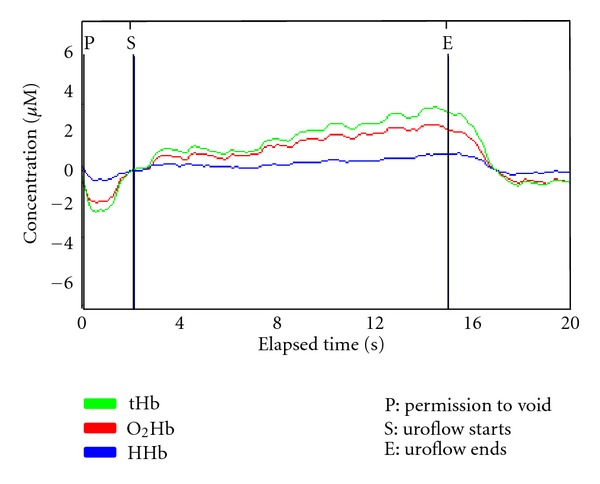
An overlaid graph of NIRS parameters zeroed to S (uroflow start) in an asymptomatic 43-year-old man. An increase in total hemoglobin (tHb) is seen as uroflow starts due to an increase in oxygenated hemoglobin concentration (O_2_Hb); this trend continues throughout voiding while HHb remains stable. This implies an increase in the provision of oxygenated blood to the detrusor as the bladder empties, as occurs in healthy striated muscle during contraction.

NIRS data provides information where there is a temporary change in the physiologic state of the tissue. Several specific parameters can be measured quantitatively in brain and muscle [3], but bladder monitoring reflects change in concentration from baseline of O_2_Hb and HHb and not absolute values [11, 21] because the total Hb concentration in the field of view is not known [2, 3]. However, plotted graphical changes in the concentration of O_2_Hb and HHb and their sum tHb provide trends and patterns of change that allow variations in hemodynamics and oxygen supply and demand to be inferred. The clarity with which such changes are seen depends on how the data are displayed, the scaling of the graph selected via the software, and whether “smoothing” of data is employed. Data displayed on printouts with simultaneous UDS data record O_2_Hb, HHb, and tHb on separate lines [12]. (Figure 1) Overlaying these parameters and zeroing them to a defined starting point (usually permission to void or uroflow start) enable trends and degrees of change to be more readily visualized in our opinion (Figure 2). 

Changes in detrusor O_2_Hb, HHb, and tHb during voiding differ between subjects with a healthy detrusor and those with several forms of bladder dysfunction [6–14, 24]. It is probable that what is observed is physiologic because similar patterns of change are seen in health and disease in studies involving voluntary muscle [2–4, 23, 25–28], and the bladder responds to specific physiologic events, including the effects of oxygen debt due to hypoxia or fatigue, and reduced blood flow or ischemia, with NIRS patterns that match those seen in other tissues [8, 11, 20, 25]. 

Physiologically this is explained by the complex mechanisms that interact to alter vessel calibre and permeability, blood volume (flow), and the concentration of O_2_Hb in the microcirculation, in order to provide tissues with adequate oxygen and substrates so that neither detrimental variations in blood supply or oxygen “debt” occur in response to alterations in metabolic demand [11, 29–31]. However, in patients with pathologies that increase metabolic demand and/or impair the normal response of the microcirculation, abnormalities do occur and oxygen debt does develop. Under these circumstances during work involving contraction the functional capacity of the muscle or organ is adversely affected and symptoms of dysfunction result [2, 8, 9, 25, 28, 32]. 

During natural voiding in asymptomatic subjects, a positive trend in tHb is usually seen following permission to void that predominantly reflects a rise in O_2_Hb. A further increase in blood volume/oxygenated hemoglobin supply often occurs following the start of uroflow, and the HHb concentration is essentially unaltered between the start and end of uroflow indicating a balance of oxygen supply and demand during voiding [8–11] (Figure 2).

**Figure 3:**
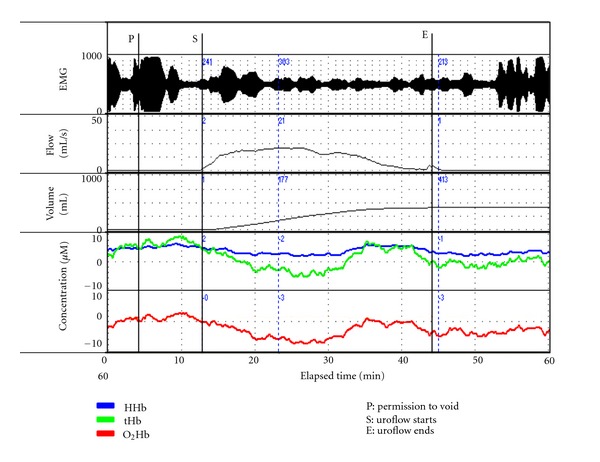
A composite graph of UDS and NIRS parameters in a 64-year-old man with LUTS associated with BOO. Following permission to void (P), an increase in total hemoglobin (tHb) occurs due to a rise in oxygenated hemoglobin (O_2_Hb). Following uroflow start (S), a marked decrease in O_2_Hb occurs reflected by a comparable fall in tHb; this trend is evident up to Qmax, and towards the end of voiding O_2_Hb rises with a corresponding increase in tHb. This implies a decrease in the provision of oxygenated blood to the detrusor during the first two thirds of the voiding cycle.

During uroflow in males with LUTS evaluated for BOO using UDS, a number of NIRS responses are seen. In the majority of those not classified as having BOO using the Abrahams Griffiths (AG) nomogram the patterns of change in NIRS parameters seen are comparable to those in asymptomatic subjects during voiding, showing some degree of positive trend in tHb and/or O_2_Hb [7, 33]. However, the predominant pattern in those diagnosed with BOO shows a negative trend in tHb often associated with a fall in O_2_Hb [14]. This negative trend (Figure 3) implies a reduced or absent hemodynamic response and/or a reduction in the availability of oxygenated blood during detrusor contraction [33]. This association led to the initial use of an algorithm to compare UDS diagnostic criteria with NIRS-derived data combined with PVR and Qmax for identifying BOO [7]. Subsequent validations by others of comparable sensitivity and specificity of NIRS-derived results [14, 17], and the effectiveness of three separate algorithms [7, 14, 33], strengthen the hypothesis that a disorder of detrusor hemodynamics is a robust physiologic rationale for LUTS in this population. Particularly as one algorithm, which is based solely on NIRS criteria (using classification and regression tree analysis), had comparable discriminant ability to the AG nomogram [33, 34]. 

**Figure 4:**
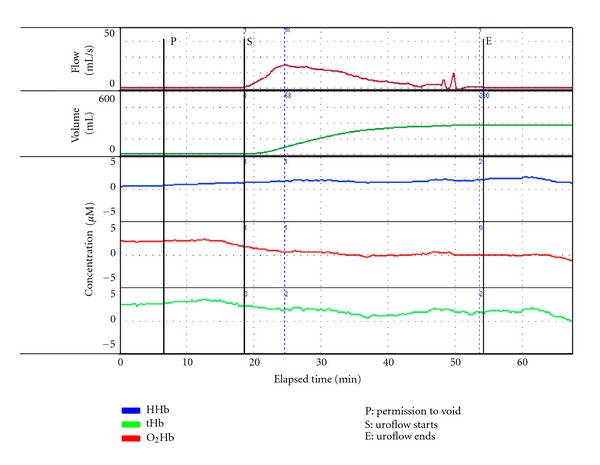
A composite graph of urine flow and volume with NIRS parameters in a 72-year-old man with obstructive lower urinary tract symptoms. He had a trabeculated bladder on cystoscopic exam and increased bladder wall thickness on ultrasound. Following permission to void (P) total hemoglobin (tHb) falls; there is a drop in oxygenated hemoglobin (O_2_Hb) associated with a rise beginning in deoxygenated hemoglobin (HHb). Following uroflow start (S), this rising (positive) trend in HHb continues while O_2_Hb and tHb continue to fall until urine flow is decreasing. This implies an imbalance in oxygen supply and demand, which begins on initiation of voiding and continues beyond Qmax.

However, other patterns of change are now evident in males with BOO. A negative trend in O_2_Hb during voiding has been observed associated with an increase in the concentration of HHb (Figure 4). This implies that an imbalance in oxygen supply and demand occurs in the detrusor under these circumstances. And in some instances, the patterns of change in O_2_Hb and HHb are effectively equal and opposite implying that significant oxygen debt has developed, as is seen in voluntary muscle that becomes fatigued during exercise [1, 8, 9, 11, 25, 35]. 

Hence patterns of change in NIRS parameters suggest an exploratory hypothesis that in some subjects with BPH and BOO the pathophysiological cause underlying their LUTS is probably detrusor dysfunction due to an abnormal hemodynamic response or the onset of oxygen debt during voiding.

## 4. Limitations 

The reproducibility of data from NIRS bladder studies is an issue that needs to be addressed in future studies. It is important to recognize what the technique does and does not measure and that each void in each patient has unique elements, which complicates even intrapatient correlation. However, characteristic patterns of change and/or positive or negative trends in O_2_Hb and HHb, and tHb associated with specific voiding events, have been identified in cohorts of patients with conditions such as BOO, DO, OAB, and NLUTD. And good statistical correlations between NIRS data and parameters identified via UDS (diagnosis of BOO, OAB, and DO) have been reported [7, 11, 13, 14, 33].

Most studies to date have sought to correlate NIRS changes in O_2_Hb and HHb, and tHb with UDS study data in patients evaluated for two conditions (OAB or BOO). However, as outlined in the introduction the parameters NIRS monitors are very different to those measured during UDS, and in both BOO and OAB as in other conditions LUTS are recognized to occur due to several different causal aetiologies [15, 16]. Hence while data can be expected to correlate in cohorts of comparable patients where LUTS are associated with disordered detrusor oxygenation or hemodynamics, consistency will not be found where different voiding pathophysiology exists that does not lend itself to detection using NIRS. 

Where NIRS data are likely to be consistent is in series of patients with comparable aetiology where altered detrusor oxygenation and/or hemodynamics underlie their LUTS. Here characteristic trends and patterns of change in NIRS parameters are likely to be evident, although interpatient variation should also to be expected due to differences in the nature of each individual's disease and the extent of their voiding dysfunction—just as occurs when UDS data are compared. 

Data in individual subjects also have potential importance. It is evident from published trends in O_2_Hb and HHb, and tHb associated with abnormal detrusor haemodynamics or oxygenation, or characteristic patterns of change occurring with defined pathology such as BPH causing BOO, that NIRS parameters have the potential to add new insights into the pathophysiology underlying symptoms of voiding dysfunction in individual patients [5, 11]. 

Movement artefact has been stated to be a limitation of NIRS [36]. However, spontaneous movement will generate abrupt simultaneous unidirectional change in all data channels and is readily recognizable, and interpretable changes of physiologic relevance can follow or precede such events. Abdominal straining may cause drift of the data streams effectively resetting the baseline but again the relevance of subsequent changes can be interpreted in relation to the “new” baseline. Muscle contraction evident on EMG may add an element of “noise”, however, even in this circumstance when physiologic changes in the data occur (when NIRS parameter trends differ), such changes can still contribute novel information to the evaluation of the patient. These facts are not recognized by some NIRS users. 

One area for focus in future studies is consistent monitoring of data of good quality—to date data sets deemed unclear (often because of noise or what is thought to be movement artefact) are being excluded from analysis [6, 7, 12, 13]. NIRS monitoring does involve a learning curve, with data quality improving with experience. How data is displayed for analysis also influences the ability to see and interpret changes of physiologic relevance. This applies particularly to having adequate scale adjustment for the NIRS data streams, and in our experience overlaying the O_2_Hb and HHb, and tHb tracings and zeroing them to a defined starting point adds clarity to interpretation [9, 10].

## 5. Discussion

NIRS monitoring can be done simultaneously with UDS pressure flow studies. The noninvasive optical methodology described for studying the detrusor is based on established principles and a body of research in other organs and tissues, as is the methodology explained for interpreting the potential physiologic relevance of NIRS data. Simultaneous NIRS and UDS studies done to date, additional urologic research using wireless NIRS [9–11, 24], and the body of NIRS studies in muscle [2–4, 23, 25–28] offer urologists an opportunity to evaluate where abnormal detrusor hemodynamics or oxygen supply and demand underlie voiding dysfunction due to BPH. This novel information has the potential to add to greater understanding of the pathophysiology underlying male LUTS because this is a condition which is recognized to be a heterogeneous clinical syndrome that probably has multiple causes of which BOO is only one [15, 16]. 

Also, it is known that the structure and vascular supply of the detrusor muscle can be altered by disease, and both have been identified as plausible sites of action by current therapeutic interventions in BPH [15, 16, 18, 37]. Hence, the exploratory hypothesis that abnormalities in detrusor hemodynamics and/or oxygen supply and demand underlie the LUTS experienced by some patients has obvious relevance in the context of both patient evaluation and potential treatment. NIRS data could contribute to the selection of therapeutic options in the future and also allow evaluation of their efficacy, as newer data indicate that the therapeutic effects of *α*_1_-Adrenoceptor antagonists, 5 *α*-reductase inhibitors, and phosphodiesterase inhibitors include direct effects on blood vessels and/or smooth muscle of the urinary bladder [6, 16, 38].

We recognize limitations in the methodology we report and that only selected cases are used to illustrate our exploratory hypothesis. NIRS is recognized to have technical limitations, and the reproducibility of data obtained in studies involving any tissue, including the bladder, is often questioned. However, objective review of the potential of this technology requires consideration of seminal publications from indexes other than Medline (e.g., Chemical Abstracts, SPIE Digital Library), and attention to the methodology used; appropriate studies should be compared, and especially not ones using different algorithms [19]. Three NIRS-derived algorithms exist related to BOO—all three have comparable levels of concordance with pressure flow studies [7, 14, 17, 34]. It is also important to recognize that the ability of this technique to contribute physiologically is limited to detection of change in the concentration of oxygenated and deoxygenated hemoglobin in the microcirculation, and that data collected from patients with heterogeneous pathophysiology can be expected to differ. However, where an underlying pathology has altered the nature of the detrusor muscle or impaired the function of the bladder's microcirculation sufficiently, NIRS monitoring can identify where perfusion of the organ with blood or the availability of oxygen during the muscular work performed during voiding is abnormal. Consequently, where such data are available, NIRS can contribute to better understanding of causal pathology by identifying where LUTS relate to abnormalities in oxygenation and hemodynamics.

Pressure flow studies remain central to the evaluation of LUTS suggestive of BPH [6, 17, 19, 39], as they are where symptoms are due to other causes. But because the function of the detrusor muscle depends on its vasculature and perfusion [36], the multiple causes that underlie LUTS likely include abnormalities of detrusor oxygenation and hemodynamics, and hence NIRS monitoring also has relevance. If studied further, NIRS can, “by assessing the hemodynamic and metabolic component of voiding, provide insight into the etiology and pathophysiology of LUTS that cannot be learned from pressure flow studies.” [6] In addition to being uniquely placed to contribute such information, this technology is also attractive because of its noninvasive nature, ability to monitor the detrusor in real time, and the feasibility of simultaneous monitoring during pressure flow studies.
